# Efficacy of PI-RADS in prebiopsy prostate-MRI at a urological cancer centre: a comparison with histology

**DOI:** 10.1186/1470-7330-15-S1-P41

**Published:** 2015-10-02

**Authors:** MD Patel, B Rangarajan

**Affiliations:** 1 The Royal Wolverhampton NHS Trust, Wolverhampton, UK

## Aims

The European Society of Urogenital Radiology (ESUR) prostate imaging-reporting and data system (PI-RADS) standardises reporting of multiparametric (MP) prostate cancer MRI. At our uro-oncology centre there has been a shift to using PI-RADS/MP MRI prior to transrectal ultrasound-guided biopsy (TRUSGB). We aim to assess the efficacy of using PI-RADS in targeted TRUSGB.

## Methods

A retrospective review was performed on 50 consecutive patients who underwent prostate MRI and subsequent TRUSGB between January-March 2015. Data were collected from MRI reports/PI-RADS to score lesion level of suspicion and location, which was correlated to Gleason grading from histology obtained through TRUSGB. Analysis and basic statistics were performed.

## Results

Histology was positive for high-grade cancer in 27/50 patients. Lesions deemed to be suspicious for cancer (PI-RADS score 4 and 5) had a positive predictive value of 83% (25/30), and were located correctly in 88%. Lesions deemed to be benign (PI-RADS score 1/2) had a negative predictive value of 80% (8/10). Equivocal lesions (PI-RADS score 3) were histologically higher grade (Gleason 3+4 and greater) in 30% (3/10), Gleason 3+3 in 10% (1/10) and negative in 60% (6/10). The overall sensitivity/specificity was 93%/62% respectively.

## Conclusion

In this sample of patients, the use of PI-RADS on prebiopsy prostate MRI has shown to have a high sensitivity and high positive predictive value in detecting/localising prostate cancer, which makes it a useful tool for targeting biopsy and detection. Going forward the high sensitivity would also have implications on the more selective use of TRUSGB.

